# Transcriptomic Analysis of Liver Indicates Novel Vaccine to Porcine Reproductive and Respiratory Virus Promotes Homeostasis in T-Cell and Inflammatory Immune Responses Compared to a Commercial Vaccine in Pigs

**DOI:** 10.3389/fvets.2022.791034

**Published:** 2022-03-24

**Authors:** Damarius S. Fleming, Laura C. Miller, Jiuyi Li, Albert Van Geelen, Yongming Sang

**Affiliations:** ^1^USDA, Agricultural Research Service, Beltsville Agricultural Research Center, Animal Parasite Disease Laboratory, Beltsville, MD, United States; ^2^USDA, Agricultural Research Service, National Animal Disease Center, Virus and Prion Research Unit, Ames, IA, United States; ^3^Department of Agricultural and Environmental Sciences, College of Agriculture, Tennessee State University, Nashville, TN, United States; ^4^USDA, National Animal Disease Center, Center for Veterinary Biologics: Policy, Evaluation and Licensing, Ames, IA, United States

**Keywords:** Porcine Reproductive and Respiratory Syndrome Virus, liver, vaccine, transcriptome, immunity

## Abstract

One of the largest impediments for commercial swine production is the presence of Porcine Reproductive and Respiratory Syndrome Virus (PRRSV), a devastating RNA viral infection that is responsible for over $1 billion in loss in the U.S. annually. The challenge with combating PRRSV is a combination of the effect of an extraordinary rate of mutation, the ability to infect macrophages, and subversion of host immune response through a series of actions leading to both immunomodulation and immune evasion. Currently there are a handful of commercial vaccines on the market that have been shown to be effective against homologous infections, but struggle against heterologous or mixed strain infections. However, vaccination is the current best strategy for combating PRRSV, making research into new vaccine technology key. To address these issues with PRRSV and host antiviral functions a novel modified-live vaccine (MLV) able to stimulate known antiviral interferons was created and examined for its ability to potentiate effective immunity and better protection. Here, we examine gene expression in the liver of pigs vaccinated with our novel vaccine, given the liver's large role in antiviral responses and vaccine metabolism. Our study indicated that pigs administered the novel vaccine experience homeostatic gene expression consistent with less inflammation and T-cell depletion risk than pigs administered the commercial vaccine.

## Introduction

Growing populations can only be sustained by the availability of healthy, abundant, and affordable food. Safeguarding these supplies requires maintaining herds in the face of established and emerging pathogens. One of the largest impediments to this for commercial swine production is Porcine Reproductive and Respiratory Syndrome Virus (PRRSV), a devastating RNA viral infection that is responsible for over $1 billion in loss in the U.S. annually (1, 2). The virus exists as two separate types, PRRSV-1 and PRRSV-2, originally named for the geographic regions in which they originated (3). The challenge with combating PRRSV is a combination of the effect of an extraordinary rate of mutation, the ability to infect macrophages, and subversion of host immune response through a series of actions leading to both immunomodulation and immune evasion (4, 5). PRRSV's ability to suppress interferon signaling compromises the antiviral response. Available commercial vaccines protect animals against homologous infections but provide less protection than heterologous or mixed strain infections (6, 7). Nonetheless, vaccination remains the best available strategy for combating PRRSV. Research into new vaccine technology may offer tools to offset anticipated viral adaptation to evade the host immune system, given the selective pressures arising from widespread vaccine use throughout the swine industry. To address these issues with PRRSV and host antiviral functions, we evaluated a novel modified-live vaccine (MLV) able to stimulate known antiviral interferons for its ability to potentiate effective immunity and better protection. Clinical studies of the vaccine prototype showed efficacy comparable to a commercially available PRRSV vaccine. Here, we examine gene expression in the liver of unvaccinated and vaccinated pigs treated with our novel or commercial MLV vaccine. The porcine liver plays a role in the ability to mount an immune response against pathogens, is affected by the vaccine's metabolism, and is large source of monocytic cells at various stages of the porcine life cycle. Monocytic cells in the form of macrophages are the cells which PRRSV infect, and though the liver is a substantial producer of monocytic cells, very little is known if the organ acts as a reservoir of the virus (8–10).

## Materials and Methods

### Vaccine Design

Our work was governed by the National Institutes of Health guide for the care and use of Laboratory animals (NIH Publications No. 8023, revised 1978). The pCMV-P129 infectious cDNA clone was constructed from the virulent PRRSV-2 (Linage 8) field virus P129, isolated in Indiana in 1995. The infectious clone pKermit (Zoetis) (4) contains the GFP gene within an additional dedicated sgRNA expression cassette. The GFP gene was replaced with genes encoding a cohort of optimized antiviral interferons (IFNs) of different sub-types. The IFN cohort-expressing virus (PRRSV-P129-IFNmix) was examined with and without an adjuvant whilst being compared to the commercial Ingelvac PRRS® ATP (MLV-ATP) MLV vaccine. The original vaccine challenge study used outbred pigs (5-week-old, *n* = 10/group).

### Experimental Design, Animals, and Sample Collection

This study design employed four of the eight treatment groups used in the clinical study of the vaccines effectiveness to protect pigs infected with the NADC-34 strain of PRRSV. The four treatments were: (a) commercial PRRSV vaccine Ingelvac PRRS® ATP (MLV-ATP), (b) ARS-novel vaccine (MLV-129), Sham-challenge (positive control), and Sham-No challenge (Negative control). Control groups were given a sham vaccination of cell culture medium at the beginning of the clinical study in concert with the vaccine treatment groups (-42 days pre-infection); all groups were challenged with a PRRSV dose of 2 ml/pig IM at 1 ×10^4^ TCID50/ml (except the negative control group which was given a sham challenge of cell culture medium). A total of 5 pigs (*n* = 5/group) were randomly selected from the four treatment groups at 14 days post infection (dpi) and had liver tissue harvested for transcriptomic analysis. This timepoint coincided with the end of the vaccine trial. Vaccinated animals were treated with 2 ml/pig of either the commercial or novel vaccine, which was administered through intramuscular shot. Pigs in all treatment groups were monitored for symptoms of illness pre and post inoculation. All animals were monitored for temperature daily and was compiled weekly. Animal weight was also observed and recorded weekly. Additional evaluations of the treatment of all treatment groups were examined and recorded daily for temperature (°C) using a subdermal chip. The threshold for determining if an animal was experiencing a febrile response was set at 40°C (104 F°). Animals were examined weekly for individual weight gain. Weight was measured in pounds and values averaged within treatment group over the time course of the study. Serology was performed on serum samples by ELISA to measure the presence of host antibodies in response to the PRRS viral challenge. Detection was based on mean S/P ratio within treatment groups for days−42 (vaccination), 0 (challenge), and 14 (necropsy). Positive antibody response was based on S/P ratios ≥0.4 as the cut-off.

### Sequencing, Quality Control, and Mapping

Representative samples of liver tissue were extracted from several locations within the liver and combined at 14 dpi. Per the method described in Fleming et al. (5) total RNA was extracted, and quality was measured using an Agilent bioanalyzer (RIN 3.4-9). Library preparation and sequencing were performed by the Iowa State University Genomics center using the 3′ Quantseq fwd kit and the Illumina Hiseq 4000 to produce twenty 100 bp single-end reads. We analyzed sequences using the following tools present at usegalaxy.org (11) and following the method used in Fleming et al. (5) with the following changes and software updates. Quality control was performed using fastp Galaxy Version 0.19.5+galaxy1 and FastQC (12) to examine raw read data, remove adapters, and reads with a phred score below 30. Alignment to the Sscrofa 11.1 reference genome was carried out using Hisat2 version 2.1.0+galaxy5 (13) set to default parameters for forward strand alignment. Raw counts were generated using FeatureCounts (14) and the Ensembl Sscrofa11.1.104 GTF file. The FeatureCounts tool was set to default parameters. Differential gene expression (DEG) was identified using DeSeq2 version 2.11.40.6 (15). The parameters were set to use poscounts for the estimate SizeFactors to account for genes with zero counts and the fit type was set to local with all other parameters tuned to default. We sought evidence for differentially expressed genes (DEGs) with a false discovery rate (*FDR)* ≤ *0.1* and with no minimum threshold for log2fc up or down regulation. Gene ontology (GO) and Pathway analysis were conducted using software from gProfiler (16) and used an FDR of ≤ 0.05 to establish significance. We also used annotation and pathway information from homologous human genes in Ensembl, NCBI, Uniprot, and KEGG (17–20).

## Results

### MLV-129p-IFNmix vs. Sham-PRRSV (Vaccinated and Challenged Groups vs. Non-vaccinated and Challenged)

**Figure 1:**
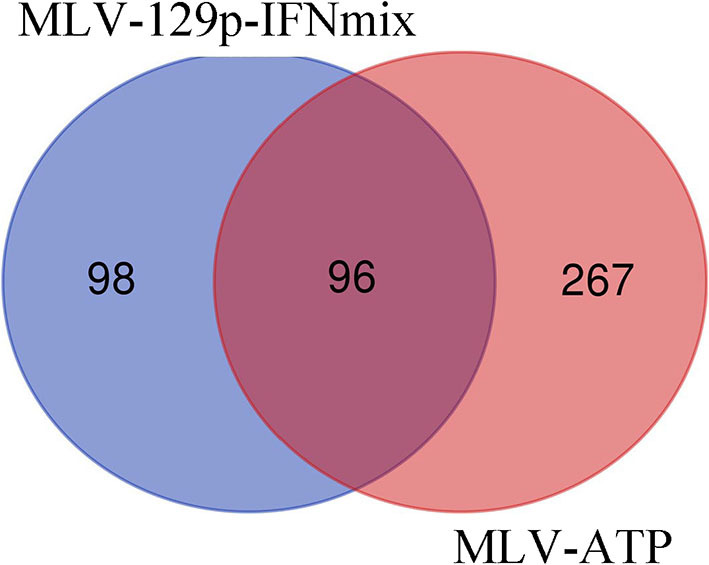
Venn diagram of statistically significant gene lists for MLV-129p-IFNmix and MLV-ATP vs. Sham-PRRSV. Genes were considered significant with FDR of ≤ 0.1.

Analysis of the MLV-129p-IFNmix vaccinated pigs vs. non-vaccinated pigs (Sham-PRRSV) at 14 dpi yielded a total of 197 differentially expressed genes (122 upregulated in the vaccinated pigs, 75 downregulated) in the liver after statistical cut-off was (FDR ≤ 0.1) applied (Supplementary Table 1). Transcriptomic analysis of the commercial vaccine (MLV-ATP) vs. the Sham-PRRSV group returned a total of 363 DEGs (187 upregulated, 176 downregulated) (Supplementary Table 2). Venn diagram analysis of the DEGs list for both vaccine groups displayed only a moderate number of overlapping genes (*n* = 96), however the number of uniquely differential expressed differed greatly between the vaccinated animals; giving the first indication that the vaccines diverged in molecular impact on the host liver (Figure 1).

### Differential Gene Expression Involved in Biological Pathways of Vaccine Treatment Groups

Examination of the list of genes being differentially expressed in the commercial MLV-ATP revealed observations related to the host response to PRRSV. The over-represented genes showed that the commercial vaccine stimulated acute inflammatory response (GO:0006954): nine of fourteen such genes were upregulated. Additionally, many upregulated genes were ascribed to the cellular response to stress (*n* = 24) (GO:0033554), homeostatic process (*n* = 26) (GO:0042592), and a human disease phenotype term Abnormal homeostasis (HP:0012337) (*n* = 31) (Table 1). These results were unique to MLV-ATP among the statistically significant terms present between both vaccinated groups. Analysis of the genes list of being differentially expressed in the MLV-129p-IFNmix also elucidated this vaccine's impact on viral immune response to PRRSV in the liver. The MLV-129p-IFNmix group included fewer differentially expressed genes related to inflammation and stress: 6 such genes are related to the human disease phenotype term increased inflammatory response (HP:0012649). The MLV-129p-IFNmix displayed a total of *n* = 8 genes involved in the cellular response to stress (REAC:R-SSC-2262752), however, three of these genes were unannotated loci. The MLV-129p-IFNmix treated group also displayed more GO terms related to negative regulation of immune response pathways, while also displaying differential expression of multiple T-cell and macrophage related pathways. The GO terms for MLV-129p-IFNmix included differential expression across innate, humoral, and adaptive immunity which was not observed with the group treated with the commercial vaccine (Table 2).

**Table 1:** MLV-ATP GO terms related to immunity, stress, and homeostasis in porcine liver.

**Term name**	**Term ID**	**FDR**
Response to stress	GO:0006950	6.18E−10
Response to virus	GO:0009615	1.53E−07
Positive regulation of cytokine production	GO:0001819	1.85E−07
Oxoacid metabolic process	GO:0043436	1.89E−06
Apoptotic process	GO:0006915	2.10E−06
Adaptive immune response	GO:0002250	1.55E−05
ISG15 antiviral mechanism	REAC:R-SSC-1169408	2.59E−05
Leukocyte mediated immunity	GO:0002443	2.88E−05
Negative regulation of viral process	GO:0048525	3.50E−05
Antiviral mechanism by IFN-stimulated genes	REAC:R-SSC-1169410	4.48E−05
Lymphocyte mediated immunity	GO:0002449	7.48E−05
Interferon signaling	REAC:R-SSC-913531	1.72E−04
Th17 cell differentiation	KEGG:04659	1.80E−04
Neutrophil extracellular trap formation	KEGG:04613	2.28E−04
T cell activation	GO:0042110	3.10E−04
Innate immune response	GO:0045087	3.60E−04
Complement and coagulation cascades	KEGG:04610	4.03E−04
ISG15-protein conjugation	GO:0032020	4.29E−04
Th1 and Th2 cell differentiation	KEGG:04658	7.49E−04
T Cell mediated immunity	GO:0002456	1.29E−03
Inflammatory response	GO:0006954	1.39E−03
Phagosome	KEGG:04145	1.63E−03
Interleukin-17 production	GO:0032620	2.18E−03
Alpha-Beta T cell activation	GO:0046631	2.44E−03
Abnormal homeostasis	HP:0012337	3.68E−03
Inflammatory mediator regulation of TRP channels	KEGG:04750	6.64E−03
Homeostatic process	GO:0042592	8.20E−03
Natural killer cell mediated cytotoxicity	KEGG:04650	2.45E−02
Leukocyte homeostasis	GO:0001776	2.69E−02
Inflammatory cell apoptotic process	GO:0006925	3.40E−02
Cellular response to stress	GO:0033554	3.72E−02
Neutrophil homeostasis	GO:0001780	3.87E−02
Viral protein interaction with cytokine and cytokine receptor	KEGG:04061	4.05E−02
Neutrophil degranulation	REAC:R-SSC-6798695	4.97E−02

**Table 2:** MLV-129p-IFNmix GO terms related to immunity, stress, and homeostasis in porcine liver.

**Term name**	**Term ID**	**FDR**
Oxoacid metabolic process	GO:0043436	1.82E−04
Negative regulation of interleukin-2 production	GO:0032703	3.59E−04
Negative regulation of T cell activation	GO:0050868	6.88E−04
Regulatory T cell differentiation	GO:0045066	7.47E−04
Immune system process	GO:0002376	1.32E−03
Biological oxidations	REAC:R-SSC-211859	1.33E−03
Macrophage migration	GO:1905517	1.98E−03
Regulation of immune system process	GO:0002682	3.01E−03
Negative regulation of T cell proliferation	GO:0042130	3.18E−03
Negative regulation of leukocyte activation	GO:0002695	3.18E−03
Regulation of interleukin-2 production	GO:0032663	4.08E−03
Interleukin-2 production	GO:0032623	4.08E−03
Complement and coagulation cascades	KEGG:04610	4.33E−03
Increased inflammatory response	HP:0012649	4.61E−03
Regulation of regulatory T cell differentiation	GO:0045589	5.12E−03
Phagosome	KEGG:04145	5.96E−03
Negative regulation of leukocyte proliferation	GO:0070664	7.03E−03
Regulation of macrophage migration	GO:1905521	7.79E−03
CD4-positive, CD25-positive, alpha-beta regulatory T cell differentiation	GO:0002361	9.78E−03
MAPK cascade	GO:0000165	1.43E−02
Negative regulation of immune effector process	GO:0002698	1.43E−02
Negative regulation of humoral immune response	GO:0002921	1.48E−02
T cell proliferation	GO:0042098	1.93E−02
Negative regulation of lymphocyte differentiation	GO:0045620	2.07E−02
Regulation of T cell activation	GO:0050863	2.36E−02
Regulation of immune response	GO:0050776	2.57E−02
Response to stress	GO:0006950	2.95E−02
Neutrophil extracellular trap formation	KEGG:04613	2.97E−02
Cellular responses to stress	REAC:R-SSC-2262752	3.51E−02
T cell activation	GO:0042110	3.64E−02
Positive regulation of MAPK cascade	GO:0043410	3.66E−02
MHC class II antigen presentation	REAC:R-SSC-2132295	4.13E−02
Complement component C5a signaling pathway	GO:0038178	4.32E−02
Mononuclear cell proliferation	GO:0032943	4.32E−02
Lymphocyte proliferation	GO:0046651	4.32E−02
Regulation of innate immune response	GO:0045088	4.96E−02

### Unique Genes and Pathways of Interest Expressed by MLV-129p-IFNmix

We endeavored to characterize the immune response in the livers of challenged pigs who had been vaccinated with a novel PRRSV vaccine. The Venn diagram depicted that there were 98 genes uniquely differentially expressed in pigs administered MLV-129p-IFNmix. We explored gene function using porcine and human annotations to determine key up and down regulated genes of biological interest (Table 3). Many of the genes in the list are natively expressed in uninfected porcine liver and lung at detectable levels (18). Some key upregulated genes of interest included the most overexpressed gene in the MLV-129p-IFNmix analysis, CD209 molecule (*CD209*), a known receptor for PRRSV (9, 21). The gene is an innate and adaptive immunity C-type lectin receptor involved in recognizing various viral pathogens, regulating virus life cycles, and promoting T-cell proliferation. In addition to *CD209*, a small group of other CD markers, CD9 molecule (*CD9*) and CD302 molecule (*CD302*) were also upregulated as a cluster unique to MLV-129p-IFNmix (18, 22). Other genes of biological interest overexpressed in the liver included cytochrome P450 family 2 subfamily R member 1 (*CYPR2R1*) a gene with ubiquitous porcine liver and lung expression that in humans is known to act as a catalyst for drug metabolism and human Vitamin D metabolism (18, 22). Some of the genes of interest interact with the host immune system through anti-inflammatory, macrophage, and immune effector actions. Genes falling into this group included TAFA chemokine like family member 4 (*TAFA4*), which has immunoregulatory activity involved in macrophage chemotaxis and increased ROS release; acid phosphatase 5, tartrate resistant (*ACP5*) a negative regulator of inflammatory responses, *IL-1B*, and *IL-12*; killer cell lectin like receptor G1 (*KLRG1*) a killer and T cell inhibitor involved in innate and adaptive immune system immunoregulatory action; and MHC class II, DO beta (HLA-DOB) an antigen presenting negative regulator and positive T-cell regulator in humans (18, 22). The study also produced downregulated genes of interest; however, they were fewer in number. Some of the downregulated genes have a lower natural expression in uninfected pigs due to liver tissue specificity, however even native genes involved in normal host functioning were observed to be down regulated such as fatty acid desaturase 1 (*FADS1*) involved in the metabolism of inflammatory lipids and T-cell cytokine production (18, 22) which may possibly link to liver metabolism effects. Other genes of interest included: lymphocyte activating 3 (*LAG3*) an inhibitory immune receptor that is part of the MHC class II adaptive immune system responses. The effect of this gene is far-reaching and may prove to be of great interest due to its involvement in negative regulatory actions. Other genes of interest included doublecortin like kinase 1 (*DCLK1*) involved in viral responses and is a negative regulator of protein localization to the nucleus in humans and alpha kinase 2 (*ALPK2*) involved apoptotic regulation and mostly cardiac related functions, but also a negative regulator of the WNT signaling pathway (18, 22).

**Table 3:** Unique genes differentially expressed by MLV-129p-IFNmix.

**Ensembl ID**	**Gene name**	**Description**	**LOG_2_FC**
ENSSSCG00000013579	CD209	CD209 molecule	1.44
ENSSSCG00000000660	A2M	Alpha-2-macroglobulin	1.00
ENSSSCG00000037762	TBX1	T-box transcription factor 1	0.94
ENSSSCG00000021724	KIF19	Kinesin family member 19	0.94
ENSSSCG00000013389	CYP2R1	Cytochrome P450 family 2 subfamily R member 1	0.88
ENSSSCG00000032282	ACP5	Acid phosphatase 5, tartrate resistant	0.86
ENSSSCG00000023498	HSPB6	Heat shock protein family B (small) member 6	0.81
ENSSSCG00000033199	TAFA4	TAFA chemokine like family member 4	0.81
ENSSSCG00000011609	FBLN2	Fibulin 2	0.79
ENSSSCG00000040461	CDKN1C	Cyclin dependent kinase inhibitor 1C	0.79
ENSSSCG00000000663	KLRG1	Killer cell lectin like receptor G1	0.77
ENSSSCG00000004596	LIPC	Lipase C, hepatic type	0.75
ENSSSCG00000015716	MARCO	Macrophage receptor with collagenous structure	0.73
ENSSSCG00000001459	HLA-DOB	MHC class II, DO beta	0.73
ENSSSCG00000001131	BTN2A2	Butyrophilin subfamily 2 member A2	0.68
ENSSSCG00000022230	CD9	CD9 molecule	0.60
ENSSSCG00000036050	CD302	CD302 molecule	0.54
ENSSSCG00000015413	FGL2	Fibrinogen like 2	0.56
ENSSSCG00000028855	GMPS	Guanine monophosphate synthase	−0.32
ENSSSCG00000004918	ALPK2	Alpha kinase 2	−0.47
ENSSSCG00000033919	DCLK1	Doublecortin like kinase 1	−0.47
ENSSSCG00000039770	SLC6A9	Solute carrier family 6 member 9	−0.69
ENSSSCG00000000688	LAG3	Lymphocyte activating 3	−0.76
ENSSSCG00000007748	PSPH	Phosphoserine phosphatase	−0.77
ENSSSCG00000027302	PKP3	Plakophilin 3	−0.79
ENSSSCG00000024015	FADS1	Fatty acid desaturase 1	−0.84
ENSSSCG00000005713	PLPP7	Phospholipid phosphatase 7 (inactive)	−0.84

*There was a total of 98 differentially expressed genes that were unique to the MLV-129p-IFNmix group. The table represents some of the genes that may be key to understanding the effects of the novel vaccine on potential markers of drug efficacy, metabolism, and anti-inflammatory immune responses (FDR ≤ 0.1)*.

## Discussion

### MLV-129p-IFNmix May Hasten Complement and Adaptive Immune Activation to PRRSV Infection Through Humoral-Mediated Expression

The results indicated interplay between MLV-129p-IFNmix and unique DEGs *CD209, A2M, HLA-DOB*, and other upregulated genes shared by both treatment groups involved in the complement cascade (Supplementary Tables 1, 2). The gene *CD209* acts as a C-type lectin receptor (CLR) capable of pattern-recognition of PRRSV (23). It is possible that the observed overexpression of *CD209* stimulates antigen presentation to T-cells, helping induce adaptive immune reactions. Additionally, as a lectin receptor *CD209* plays a role in the complement cascade which supports the transition to adaptive immune functions. Multiple genes involved in both innate and adaptive immune function suggest that the novel vaccine engender quicker adaptive immune system recognition, reducing the cellular and physical stress of the host-pathogen interaction (24, 25).

### MLV-129p-IFNmix Promotes Negative Regulation of Processes That May Bolster Immune Response to PRRSV

Pigs administered the novel vaccine exhibited changes in several negative regulatory processes and molecular functions (Table 2). The range of these pathways were centered around activation and proliferation of T-cells and leukocytes. The genes involved in these responses present in our study are mostly upregulated, pointing to a relaxing of the immune response characteristic of the innate arm of the porcine immune response. The preponderance of negative regulation at 14 dpi in the novel vaccine group was observable at both the pathway and gene level involving differential expression spanning both directions. The action of MLV-129p-IFNmix shows promise related to immune/T-cell exhaustion. Although T-cell exhaustion more commonly results from prolonged chronic infections, the abbreviated maturation experienced by commercial pigs could make a PRRSV infection essentially a lifelong infection. T-cell exhaustion risks depleting memory T-cells, severely handicapping the immune response. One of the first indicators of this is thought to be negative regulation of interleukin 2 (*IL-2*) (26). Here, of four genes involved in the negative regulation of interleukin-2 production (GO:0032703), three were downregulated in pigs receiving the novel vaccine (Table 2). One of these (*LAG3)* was unique to the novel vaccine's DEG list. Therefore, the novel vaccine may stimulate a better genomic-mediated immune response in the liver, reducing viral loads at 14 dpi. The gene *LAG3* is an example of this. Its downregulation may affect negative regulatory processes that include IL-2 production and Treg cell differentiation. Recent studies of human *LAG3* gene action suggest a possible role in immunomodulation and viral immune evasion (27). The downregulation around *LAG3* is intriguing when viewed from the perspective that the difference in the liver response between vaccines may involve T-cell exhaustion. The phenomenon of T-cell exhaustion is thought to result from persistent signaling during chronic viral illness and cancers, which seems to be partly driven by *LAG3* upregulation. It is possible that *LAG3* downregulation may reduce inhibition of a particular type of T-cell response to PRRSV infection in pigs vaccinated with MLV-129p-IFNmix. It may be that the downregulation of the *LAG3* inhibitory signaling keeps T-cell proliferation and activation at a metabolically sustainable level. In humans, overexpression of *LAG3* is correlated with disease progression in viral infections. Downregulation may allow for less suppression surrounding regulatory T-cells, but limit CD8 or CD4 T-cells. Another gene uniquely expressed by the novel vaccine is, fibrinogen like 2 (*FGL2)* (Table 3); it is in the same gene family as *FGL1*, a major ligand and co-activator of *LAG3*'s inhibitory action (18, 22). Porcine *FGL1* may not downregulate *LAG3* action, but rather upregulate *FGL2*, thereby negatively regulating dendritic cell, memory T-cells, and macrophage antigen processing. However, *FGL2* does support T-cell activation on MHC molecules (18, 22, 28, 29). There was also unique macrophage (M2) gene expression. Action of the gene *FGL2* on macrophages in concert with effector actions on regulatory T-cell expression has been observed to promote repair among macrophages, possibly in response to chronic illness or inflammation. The novel vaccine might improve macrophage, T-cell, and pro-inflammatory balance or may sort infected macrophages (needing opsonization or phagocytosis) from uninfected. Reduced expression may keep T-cell proliferation and activation at a metabolically sustainable level, improving the efficiency of established immune checkpoints against PRRSV.

## Conclusion

Each vaccine elicited distinct patterns of gene transcription in the liver of infected pigs. As compared to pigs administered the commercial vaccine, those administered the novel MLV-129p-IFNmix vaccine had fewer genes in flux at 14 dpi. They may therefore have been experiencing less viral-induced stress on the organ/immune system. Compared to the MLV-129p-IFNmix, pigs receiving the commercial vaccine experienced overexpression of more genes related acute inflammatory responses. Thus, pigs vaccinated with the commercial vaccine may experience a longer innate immune response to the virus, more pathology, and poorer growth. Pigs administered the commercial vaccine also showed differential expression of interferon stimulating genes (ISGs) not present in the MLV-129p-IFNmix. It is possible that by supplying interferons as part of the vaccine matrix, the experimental MLV-129p-IFNmix introduces interferon stimulation earlier than the commercial vaccine. However, more time points will need to be explored to determine when such stimulation occurs. From a transcriptomic standpoint, MLV-129p-IFNmix appears to show better cellular homeostasis in the liver. By contrast, animals administered the commercial vaccine experience less homeostasis and more metabolic and cellular stress.

## Data Availability Statement

The data discussed in this publication have been deposited in NCBI's Gene Expression Omnibus (GEO) (30) and are accessible through GEO Series accession number GSE197014 (https://www.ncbi.nlm.nih.gov/geo/query/acc.cgi?acc=GSE197014).

## Ethics Statement

The animal study was reviewed and approved by Institutional Animal Care and Use Committee at National Animal Disease Center Ames, IA.

## Author Contributions

DF: methodology, computation software, transcriptomic data validation, formal analysis, review and editing, and writing and original draft preparation. DF, LM, and AV: animal tests and sample preparations. DF and LM: investigation, resources, data curation, and supervision. DF, LM, JL, and YS: writing and original draft preparation. DF, LM, and YS: project administration. YS and LM: funding acquisition. All authors have read and agreed to the published version of the manuscript.

## Funding

This work was supported by USDA NIFA AFRI Grant 2013-67015-21236, and in part by USDA NIFA AFRI 2015-67015-23216 and USDA NIFA 2018-67016-28313. Post-doctoral funding for DF provided by the Oak Ridge Institute for Science and Education (ORISE).

## Conflict of Interest

The authors declare that the research was conducted in the absence of any commercial or financial relationships that could be construed as a potential conflict of interest.

## Publisher's Note

All claims expressed in this article are solely those of the authors and do not necessarily represent those of their affiliated organizations, or those of the publisher, the editors and the reviewers. Any product that may be evaluated in this article, or claim that may be made by its manufacturer, is not guaranteed or endorsed by the publisher.

## Supplementary Material

Supplementary Table 1 MLV-129p-IFNmix differentially expressed gene list.

Supplementary Table 2 MLV-ATP differentially expressed gene list.
